# High‐dose non‐sedating antihistamines are used insufficiently in chronic urticaria patients treated with omalizumab

**DOI:** 10.1002/clt2.12085

**Published:** 2021-12-11

**Authors:** Hanne Madsen, Charlotte G. Mortz, Carsten Bindslev‐Jensen, Mette Reilev, Jesper Hallas, Daniel P. Henriksen

**Affiliations:** ^1^ Department of Dermatology and Allergy Center Odense University Hospital Odense Denmark; ^2^ Department of Internal Medicine & Acute Medicine Odense University Hospital Odense Denmark; ^3^ Clinical Pharmacology, Pharmacy and Environmental Medicine Department of Public Health University of Southern Denmark Odense Denmark; ^4^ Department of Clinical Pharmacology Odense University Hospital Odense Denmark

**Keywords:** adherence, antihistamines non‐sedating, chronic urticaria, guideline, omalizumab

## Abstract

**Background:**

The lifetime prevalence of chronic urticaria (CU) is 0.5%–1%. In some patients with CU, symptomatic control is not achieved with non‐sedating second‐generation H1 antihistamines (nsAH1) alone, even with quadrupled standard doses as recommended in international guidelines. In these cases, biological treatment with omalizumab can be added. Since omalizumab is expensive compared to antihistamines, lack of adherence to guidelines for high dose nsAH1 (up to four‐fold standard dose per day) may be associated with substantial unnecessary costs. The aim was to measure the use nsAH1 before and during omalizumab use for the first time in an omalizumab treated CU population.

**Methods:**

We identified all Danish patients with CU who initiated omalizumab from March 2014 to December 2018 and evaluated new and ongoing nsAH1 treatments using the Danish nationwide registries.

**Results:**

A total of 955 CU patients initiated treatment with omalizumab within the study period (median age 40 years [IQR 28–50], 74.5% females). During the 12 months prior to omalizumab initiation, 95.6% of the patients filled at least one prescription with nsAH1 at some point, while 84.7% filled at least one prescription during the three months before omalizumab. From 3 months before omalizumab initiation till 3 months after, the proportions of users of high‐dose nsAH1 was maximum 31.1%.

**Conclusions:**

Omalizumab was usually administered before sufficient nsAH1 treatment was tried. In despite of the labelling that omalizumab should be co‐administered with high dose nsAH1, this does not happen This may lead to substantial unnecessary costs.

## 1 INTRODUCTION

Chronic urticaria (CU) is characterized by appearance of wheals, angioedema, or both for more than 6 weeks to known or unknown causes. CU is divided into two subtypes: chronic spontaneous urticaria (CSU) and inducible urticaria that is cold urticaria or aquagenic urticaria.^1^, ^2^, ^3^, ^4^ CU may continue for months or years. The lifetime prevalence of CU is 0.5%–1%, and the disease occurs twice as frequently among women. Generally, symptoms break out when patients are between 20 and 40 years old.^4^

A step‐wise approach has been developed for the management of CU,^1^, ^2^, ^3^, ^4^, ^5^, ^6^, ^7^ and non‐sedating second‐generation H1 antihistamines (nsAH1) are considered first‐line treatment. However, 50% of patients have inadequate response and fail to achieve complete control,^4^ even when dosing was increased up to four‐fold above standard doses of the nsAH1s bilastine, cetirizine, desloratadine, ebastine, fexofenadine, rupatadine and levocetirizine^1^, ^2^, ^3^, ^6^, ^7^, ^8^ and loratadine.^1^ This is the recommended first‐line treatment, while quadrupled nsAH1 in combination with omalizumab is second‐line treatment.^1^

Omalizumab is a monoclonal antibody directed against IgE. Since February 2014 it has been approved in Denmark for the management of CSU in patients aged >12 years who are inadequately controlled by nsAH1 in high doses.^9^

Treatment with omalizumab is considered safe and efficient.^10^, ^11^, ^12^, ^13^, ^14^, ^15^ High dose antihistamine is supported by the Danish Heath Service.^11^

Cyclosporine is third‐line treatment.^1^ Alternative therapy includes methotrexate, montelukast and short courses of systemic glucocorticoids.^1^, ^3^, ^4^, ^5^, ^6^, ^7^

Real‐world adherence to antihistamines has only been sparsely examined, but usage of antihistamine in amounts below recommendations before and after omalizumab initiation has been demonstrated.^16^ Lack of adherence to guidelines is associated with substantial unnecessary costs, as one‐year treatment with antihistamines plus omalizumab in Denmark increases expenses by more than 3.400%.^17^

## 2 AIM

The aim of this study was to examine guideline adherence to high‐dose nsAH1 before and after initiation of omalizumab for the first time in CU population.

## 3 METHODS AND MATERIALS

**FIGURE 1:**
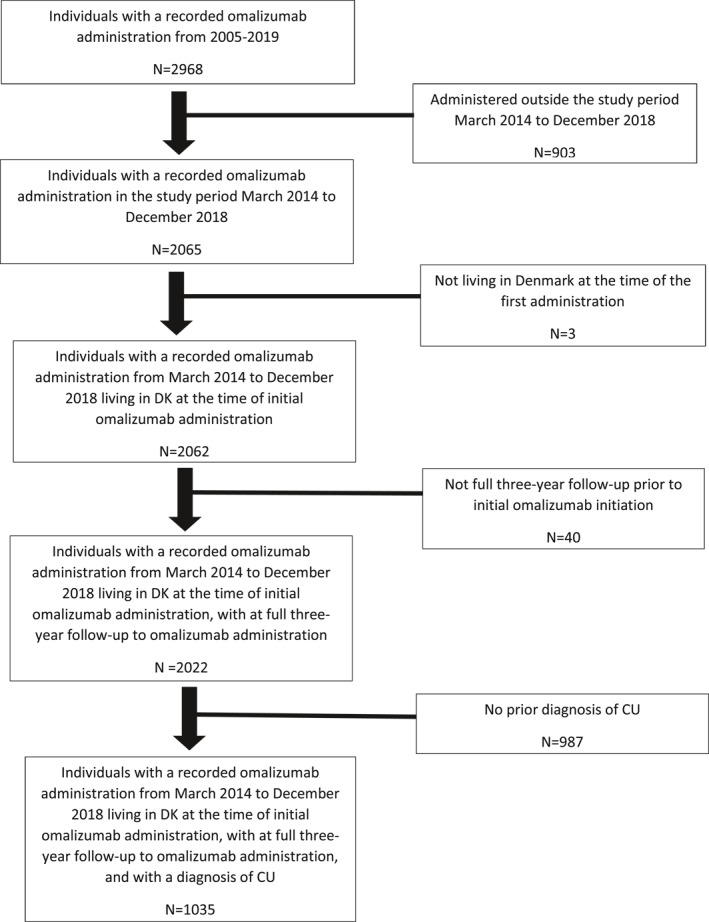
Formation of study population including patients with chronic urticaria (CU) receiving omalizumab from 1 March 2014 until December 2018

We included all patients in Denmark, who initiated treatment with omalizumab and previously was diagnosed with CU during the study period from March 2014 to December 2018 (Figure 1). We used the Danish nationwide health‐ and prescription registries to describe new drug treatments and diagnoses in the period leading up to and following initiation of omalizumab.

### 3.1 Data sources

The Danish National Health Service provides universal tax‐supported health care for all Danish residents, thereby allowing truly population‐based register‐linkage studies. We retrieved data from three Danish nationwide administrative registers: The Danish Civil Registration System,^18^ The Danish National Patient Register (DNPR),^19^ and the Danish Register of Medicinal Product Statistics (RMPS).^20^

The Danish Civil Person Register contains data on vital status (date of birth and death) and migrations to and from Denmark since 1968.^18^

The DNPR holds information for all contacts to Danish hospitals since 1977.^19^ From 1995, outpatient clinic contacts, and emergency department contacts have been included in the DNPR. Diagnoses were recorded according to International Classification of Diseases (ICD)‐10 since 1994 (Appendix 1).

The RMPS holds information on all filled drug prescriptions from community pharmacies since 1995.^20^ Prescription records data include the Central Person Registry number, date of filled prescription, the substance, brand name, and quantity. Drugs are categorized according to the Anatomical Therapeutic Chemical (ATC) code and the quantity is expressed by the use of the defined daily dose (DDD).^20^, ^21^

### 3.2 Study population

We identified all patients who initiated omalizumab in Denmark from March 2014 to December 2018 with a prior diagnosis of urticaria. This population is believed to represent severe cases of CU (Figure 1).

CU is divided into CSU and inducible urticaria: symptomatic dermographism, cold urticaria, delayed pressure urticaria, solar urticaria, heat urticaria, vibratory angioedema, cholinerg urticaria, contact urticaria and aquagenic urticaria. These are all treated according to the same treatment algorithm including omalizumab,^1^ and consequently all Danish ICD‐10 codes for inducible urticaria variants are included in the study although omalizumab for inducible urticaria in Denmark still is off label (Appendix 1).

The Charlson Comorbidity Index^22^ was used as a surrogate marker for the overall comorbidity burden and is based on ICD‐10 codes recorded from in‐ or out‐patient hospital contacts.^23^ Selected comorbidities were chosen and estimated by presence of hospital‐given diagnoses or use of relevant medications dispensed from public pharmacies in Denmark.

### 3.3 Description of drug use

In Denmark, CU treatment is initially managed by primary care physicians and dermatologists working out‐side the hospitals. If second‐line treatment with omalizumab is needed, this can only be subscribed at dermatological or pulmonary department in reference centres at tertiary hospitals.

We identified omalizumab injections using drug codes in the DNPR (BOHJ19A1). In Denmark, the hospital departments use procedure codes together with the drug code to record use of expensive drug therapy such as biologicals. This method of identification has been shown to have a high validity in other therapeutic groups.^24^

To define guideline adherence, we used the DDD as the unit of measure. The DDD represents the assumed average maintenance dose per day for a drug used for its main indication in adults. DDD is a technical unit of measurement and should not be mistaken for the recommended or the prescribed daily doses.^21^ DDDs are presented in the Appendix 2. Based on treatment recommendation, guideline adherent antihistamine use was set as 4.5 times of DDD for fexofenadine (540 mg) and four times DDD for any other nsAH1 (Appendix 2).

### 3.4 Analysis

Based on the recorded prescription data on nsAH1 we calculated average consumed daily doses in 3 months intervals from 12 months before‐till 12 after omalizumab initiation, expressed as DDDs per day. We then categorized this into low‐dose (0, 1–1.9 and 2–3.9 times) and high‐dose (4–7.9 and 8+ times) consumed DDD per day.

Categorical variables were presented as numbers and percentages, and continuous variables as medians with interquartile ranges (IQR). Computation of baseline characteristics was based on the index date that is, the date of first recorded procedure code of omalizumab administration. Using relevant ICD‐10 codes (diagnoses) and ATC codes (prescribed drugs), the prevalence proportions of selected comorbidities at the time of omalizumab initiation were calculated as well as quarterly prevalence proportions of filled prescriptions of nsAH1, sedating first‐generation antihistamines, glucocorticoids, and montelukast. In addition, the incidence rate of first nsAH1 use was charted in 1‐month intervals within 12 months before till 12 months after omalizumab initiation.

### 3.5 Ethics

Data were analysed using the framework of the Danish Health Data Board, using Stata version 17. Within this framework pseudonymized individual‐level data were available to researchers. For confidentiality reasons, reporting exact counts below 5 is not permitted. According to Danish law, approval from an ethics committee is not required for pure register‐based studies.

## 4 RESULTS

We included 955 patients treated with omalizumab with a diagnosis of CU during the study period from March 2014 till December 2018.

The majority were women (74.5%), and the median age was 40 years (IQR 28‐50) (Table 1). The prevalence of comorbidity in the population was low, and 90.0% had a Charlson Comorbidity Index below or equal to 1. The two most common comorbidities among the selected comorbidities were anxiety/depression (19.6%) and chronic rhinitis (18.3%).

**TABLE 1:** Baseline characteristics, demographics data and drug use at the time for the first omalizumab injection

Total	(*n* = 955)
Age, median (IQR)	40 (28–50)
Median time to end‐of‐follow‐up in months (IQR)	24 (12–38)
Median omalizumab treatment duration in months (IQR)	16 (6–31)
Age	
18–29	252 (26.3%)
30–44	335 (35.1%)
45–64	292 (30.6%)
65+	76 (8.0%)
Sex	
Female	711 (74.5%)
Male	244 (25.5%)
Charlson comorbidity index (CCI)	
0	636 (66.6%)
1	223 (23.4%)
2	59 (6.2%)
≥3	37 (3.9%)
Urticaria diagnosis	
Urticaria chronica	526 (55.1%)
Urticaria, unspecified	264 (27.6%)
Allergic urticaria	198 (20.7%)
Idiopathic urticaria	167 (17.5%)
Urticaria due to cold or heat	38 (4.0%)
Urticaria due to pressure	34 (3.6%)
Urticaria recidivans	29 (3.0%)
Urticaria cholinergica	20 (2.1%)
Urticaria solaris	5 (0.5%)
Urticaria aquagenica	(*n* < 5)
Urticaria vibratoria	(*n* < 5)
Selected comorbidities	
Anxiety or depression^b^	187 (19.6%)
Chronic rhinosinuitis^c^	175 (18.3%)
Obesity^d^	94 (9.8%)
Serious mental disorders^e^	79 (8.3%)
Atopic dermatitis^a^	37 (3.9%)
Food allergy^f^	14 (1.5%)
Current drug use (prescription up to 12 months prior)	
Non‐sedative antihistamines	913 (95.6%)
Montelukast	498 (52.1%)
Glucocorticoids	475 (49.7%)
Sedative antihistamines	42 (4.4%)
Methotrexate	12 (1.3%)
Ciclosporine	6 (0.6%)

^a^ ICD‐10 L20.

^b^ ICD‐10 F32, F33, F40, F41 or ATC N06A.

^c^ ICD‐10 J310, J32, J33 or ACT R01AD.

^d^ ICD‐10 E66, or ACT A084.

^e^ ICD‐10 F31, F20–29 or ACT N05A.

^f^ ICD‐10 T780, T781B.

The median length of treatment with omalizumab was 16 months (IQR 6–31 months).

Omalizumab was used for both CSU and inducible urticaria (Table 1). The three most common indications for omalizumab initiation were CU (55.1%), unspecified urticaria (27.6%), and acute or chronic allergic urticaria (20.7%) (Table 1).

During the 12‐month interval prior to omalizumab initiation, 95.6% of the patients filled a prescription on nsAH1, 52.1% on montelukast and 49.7% on glucocorticoids. Few of the included patients filled prescriptions of first‐generation antihistamines (4.4%), cyclosporine (0.6%) or methotrexate (1.3%).

**FIGURE 2:**
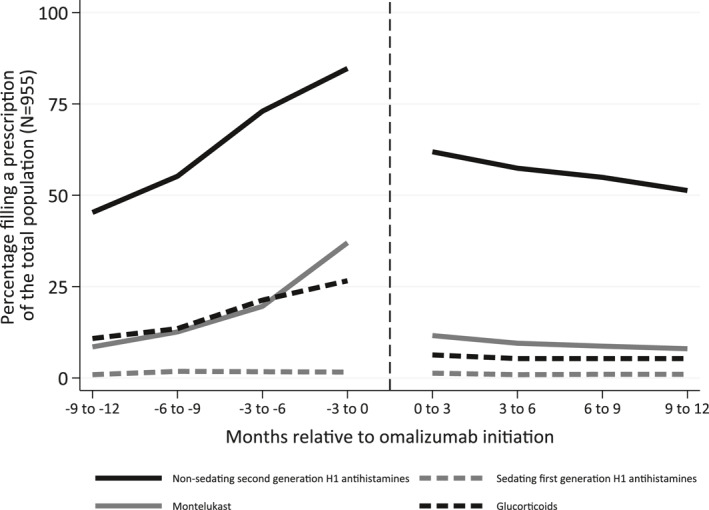
Quarterly prevalence proportion of filled prescriptions of non‐sedating second generation H1 antihistamines, sedating first generation antihistamines, montelukast, and systemic glucocorticoids one year prior to, and after omalizumab initiation

The quarterly prevalence proportion of filled prescriptions on the most common drugs against CU increased during the 12 months prior to initiation of omalizumab and dropped instantaneously afterwards. As such, we observed that approximately 80% filled a prescription on nsAH1, 35% on montelukast and 25% on glucocorticoids in the 3‐month interval immediately before omalizumab initiation. After omalizumab initiation, the prevalence proportion of both glucocorticoids and montelukast decreased to a level below 10%, while the prevalence proportion for nsAH1 decreased to 50% without reaching a plateau (Figure 2).

**FIGURE 3:**
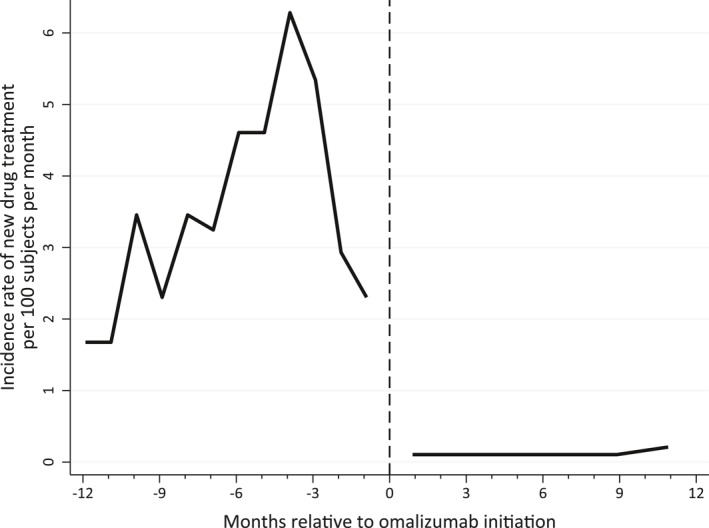
The incidence rate of non‐sedating second generation H1 antihistamines in 1‐month intervals within 12 months before to 12 months after first omalizumab injection

The incidence rate for nsAH1 was highest 3 months prior to omalizumab treatment (6.3 new users/100 patients/month), and only very few filled their first nsAH1 prescription after initiation of omalizumab (0.1 new users/100 patients/month) (Figure 3).

Thus, the majority of CU patients who used nsAH1, filled their prescription prior to omalizumab initiation.

**FIGURE 4:**
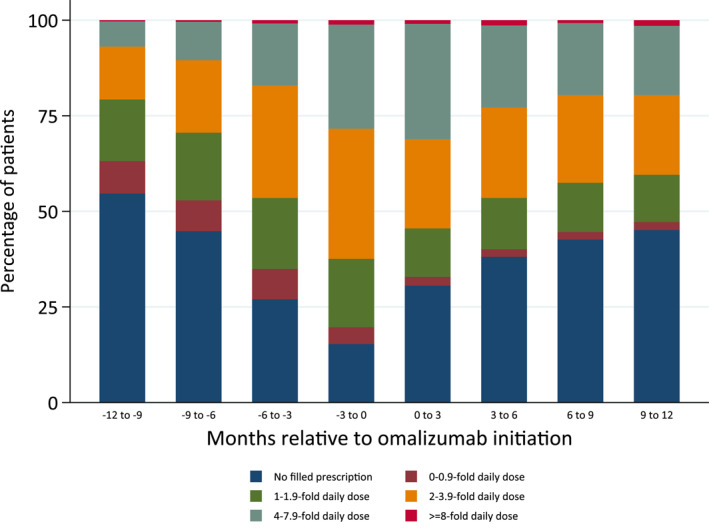
Percentage of patients filling prescriptions of non‐sedating second‐generation H1 antihistamines 12 months before and after initiation of omalizumab, categorised according to average daily intake, measured in defined daily doses per day. Calculated within 3‐month intervals, relative to the initiation of omalizumab

The dosage of filled nsAH1 varied between patients (Figure 4). A total of 271 patients (28.4%) filled prescriptions corresponding to high‐dose nsAH1 in the interval 0–3 months before omalizumab initiation, while 148 patients (15.3%) did not fill any prescription of nsAH1 during this interval. During the first 3‐month interval immediately after omalizumab initiation, 31.1% filled prescriptions corresponding to high‐dose nsAH1.This was the highest observed prevalence in any quarter (Figure 4). The percentage decreased continuously to 19.6% in the interval 9–12 months after initiation.

## 5 DISCUSSION

In this 4.5‐year nationwide longitudinal survey we investigated prescription patterns of nsAH1 one year prior to and after initiating omalizumab in patients with CU. A total of 955 patients initiated treatment with omalizumab within the study period. 12 months prior to omalizumab initiation, 95.6% of the patients filled at least one prescription with nsAH1 at some point, while initiation 84.7% filled at least one prescription three months before omalizumab. From 3 months before omalizumab initiation till 3 months after, the proportion of users of high‐dose nsAH1 increased from 28.4% to 31.1%. These were the highest prevalence proportions in any time during the observation period. Though most patients filled a nsAH1 prescription 3 months before initiation of omalizumab, less than one‐third of the patients' filled prescriptions for high‐dose nsAH1 three months before and after first omalizumab injection. In general, the dosage filled was below recommendations of high‐dose nsAH1 for at least two‐thirds of the population. Thus, it is evident, that most Danish CU patients are not treated according to guidelines.^1^, ^3^, ^25^, ^26^

Suboptimal usage of nsAH1 has also been documented in the US population receiving omalizumab in an observational cohort study queried medical and pharmacy claims data. The use of concomitant nsAH1 decreased significantly from baseline though the 1–6 and 7–12 months after omalizumab initiation, respectively 44.0% versus 33.6% versus 21.5%.^16^

A German prospective, non‐interventional study, AWARE, demonstrated, that only 3% of patients with uncontrolled CSU received up‐dosing of H1‐antihistamines and less than half of the patients received any antihistamines before and after initiating omalizumab.^27^ A later study using retrospective chart review demonstrated, that 20.4% of patients received omalizumab monotherapy without any nsAH1.^28^

Low adherence to prescribed treatment has been widely reported in patients with chronic diseases.^29^ In questionnaires among patients with CU they report compliance to oral antihistamines in only 52%^30^ and 53.2%^31^ of the dosages. So, reasons for insufficient nsAH1 dosing might be dual. Both that, the doctors are not adherent to the guideline‐recommended treatments^27^, ^28^ and patients are not adherent to the prescribed medication.^29^, ^30^

Thus, it is important to be aware of sub‐standard adherence in CU patients. Firstly, due to the patient's own inconvenience of being treated with injection and hospitals visits for a condition that might be treated with oral medicine. Secondly, tapering of omalizumab might be long lasting or be unsuccessful if the patient does not use high‐dose nsAH1.^32^ Thirdly, to avoid overspending of money, since omalizumab is expensive. In Denmark quadrupled fexofenadine 540 mg, once daily, costs approximately 1980 DDK (266 Eur/year), while the cost of omalizumab is approximately 69,600 DDK (9.355 Euro)/year used in standard dose 300 mg subcutaneous every fourth week.^17^ In Denmark, it is possible for nurses and doctors to see, which prescriptions the patient has filled at the pharmacy (https://fmk‐online.dk/fmk/), but to improve the adherence of a patient is very difficult.^29^ One of the methods could be frequent consultation.^30^ It is therefore recommended to have regular consultations with the patient. As in‐house hospitals resources are limited and meeting at hospital is inconvenient for the patient, we suggest implementing mHealth‐guided management as developed for airway diseases.^33^ This could include virtual consultations verifying adherence.^30^

The main strength of this study is the population‐based approach. The study covers the entire Danish population during a 4.5‐year period and links individual‐level data from three highly valid national registries.^18^, ^19^, ^20^ As proxies for drug utilization, we used prescription data from RMPS,^20^ which possess high data completeness and thereby minimizes the risk of information bias.

The validity of the CU diagnosis code has not been formally validated in the DNPR. However, since this is a population of omalizumab users, we can expect the CU diagnosis to be valid. The recording of omalizumab codes is likely to be valid as well. We used a DNPR procedure code to identify omalizumab dispensing. A Danish study has shown high validity when using these codes,^24^ and registration is a highly prioritized task in the Danish hospitals, since the department receives no reimbursement for this expensive treatment, if they do not use the correct codes.

We included both CSU and inducible urticaria despite of omalizumab being indicated for patients with CSU only, but sensitivity analyses showed no difference in the pattern of nsAH1 use, when limiting to patients with a CSU‐diagnosis.

During the study period at least 15.3% of CU patients did not fill in any prescriptions for nsAH1. Some NsAH1 (ebastine, cetirizine and fexofenadine 120 mg) are, however, sold over‐the‐counter. During the study period the proportion of person‐identifiable sales was 32%–36% for cetirizine, 69%–71% for ebastine, and increasing to more than 90% for the remaining nsAH1s. Since prescribed nsAH1s are reimbursed, it seems unlikely that patients would initiate over‐the‐counter nsAH1 after the start of omalizumab and over‐the‐counter sales are not expected to have a significant impact on the results.

In conclusion, this study revealed that the recommended high‐dose use of nsAH1 in urticaria patients before or during omalizumab treatment is a maximum of 31%. Thus, omalizumab was administered before sufficient nsAH1 treatment was tried. Despite of the labelling that omalizumab should be co‐administered with high dose nsAH1, this does not happen. To minimize substantial unnecessary costs, treatment guidelines should be revisited by doctors treating CU and patient adherence controlled at frequent consultations.

## CONFLICT OF INTEREST

None.

## Supporting information

Supplementary Information S1
