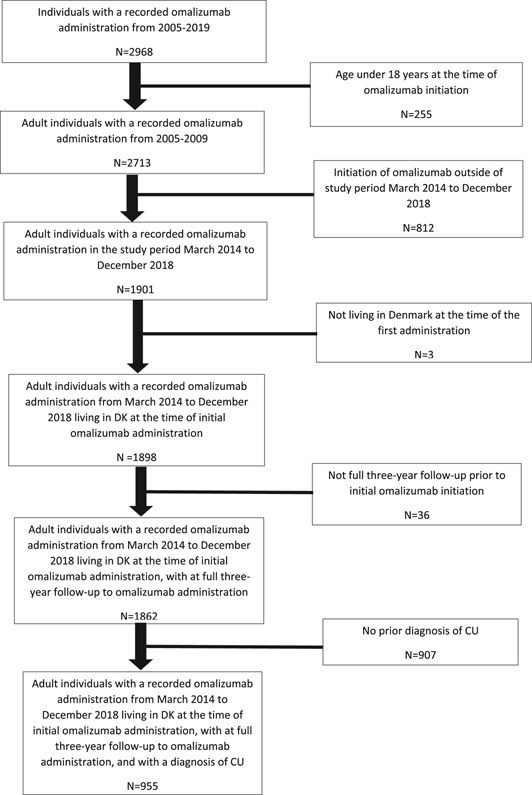# Corrigendum: High‐dose non‐sedating antihistamines are used insufficiently in chronic urticaria patients treated with omalizumab

**DOI:** 10.1002/clt2.12120

**Published:** 2022-02-08

In [1], the Figure 1 was incorrect and should have been corrected.

The correct Figure 1 should be: