# Supplementary material for: High‐dose non‐sedating antihistamines are used insufficiently in chronic urticaria patients treated with omalizumab
Source: Clin Transl Allergy. 2021 Dec 11;11(10):e12085. doi: 10.1002/clt2.12085 (PMC8665689; doi:10.1002/clt2.12085)
Supplement: Supplementary file 1 — Supplementary Information S1 [file CLT2-11-e12085-s001.docx]

**Appendix 1**

Included urticaria ICD-10 diagnosis

Chronic spontaneous urticaria (CSU)

DL500 allergic urticaria

DL501 idiopathic urticaria

DL508A urticaria chronica

DL508B urticaria recidivans

DL509 urticaria unspecified

Inducible urticaria

DL502 urticaria due to cold or heat

DL504 urticaria vibratoria

DL505 urticaria cholinergica

DL508C urticaria aquagenica

DL508E urticaria due to pressure

DL563 urticaria solaris

**Appendix 2**

Defined daily doses (DDD) for the most frequently used drugs against chronic urticaria (CU).

| ACT code | Name | DDD (mg) | Quadruple dose/DDD | Unit |
| --- | --- | --- | --- | --- |
| **Second generation**  **antihistamines** |  |  |  |  |
| R06AX22 | ebastine | 10 | 40/4 | mg |
| R06AX26 | fexofenadine | 120 | 540/4,5 | mg |
| R06AX27 | desloratadine | 5 | 20/4 | mg |
| R06AX28 | rupatadine | 10 | 40/4 | mg |
| R06AX29 | bilastine | 20 | 80/4 | mg |
| R06AE07 | cetirizine | 10 | 40/4 | mg |
| R06AE09 | levocetirizine | 5 | 20/4 | mg |
| **First generation antihistamines** |  |  |  |  |
| R06AD02 | promethazine | 25 |  | mg |
| R06AE05 | meclozine | 50 |  | mg |
| R06AA04 | clemastine | 2 |  | mg |
| R06AE03 | cyclizine | 100 |  | mg |
| N07CA02 | cinnarizine | 90 |  | mg |
| R06AA02 | diphenhydramin | 200chlorid/  300teoclate |  | mg |
| **Glucocorticoides** |  |  |  |  |
| H02AB09 | hydrocorticosone | 30 |  | mg |
| H02AB04 | methylprednisolone | 7.5 |  | mg |
| H02AB02 | dexamethasone | 1.5 |  | mg |
| H02AB01 | betamethasone | 1.5 |  | mg |
| H02AB08 | triamcinolon | 7.5 |  | mg |
| H02AB06 | prednisolone | 10 |  | mg |
| **Others** |  |  |  |  |
| R03DC03 | montelukast | 10 |  | mg |
| L04AD01 | ciclosporin | 250 |  | mg |
| L04AX03 | methotrexate | 2.5 |  | mg |
